# Use of High-Protein and High-Dietary-Fibre Vegetable Processing Waste from Bell Pepper and Tomato for Pasta Fortification

**DOI:** 10.3390/foods12132567

**Published:** 2023-06-30

**Authors:** Dorota Teterycz, Aldona Sobota

**Affiliations:** Department of Plant Food Technology and Gastronomy, Division of Engineering and Cereals Technology, University of Life Sciences in Lublin, Skromna 8, 20-704 Lublin, Poland; dorota.teterycz@up.lublin.pl

**Keywords:** durum wheat pasta, by-products, zero-waste, plant protein sources, pepper seeds, pepper placenta, tomato waste, amino acids composition

## Abstract

There is worldwide wastage of 1.3 billion tons of food annually. It is recommended that food waste should be reduced at every phase of production. By-products from food processing have high nutritional value so their use in new products is advisable. The aim of the study was to enrich the nutritional value of pasta using waste from the food industry. By-products from tomato processing (tomato waste—TW) and pepper (defatted pepper seeds—DPS, pepper placenta—PP) were used at a level of 10–30% to produce pasta. The farinographic characteristics, chemical composition, cooking quality, and colour of the pasta were studied. The results show a significant (*p* < 0.05), up to 27%, increase in the protein content of the TW30 samples, compared with the control (16.16% d.m. vs. 20.61% d.m.). The TDF content increased over five times in DPS30 and TW30 (27.99% d.m. and 25.44% d.m.). The amino acid composition of the pasta improved with the fortification but failed to achieve complete protein by FAO. The DPS30, PP20, PP30 and all TW samples can be considered high-protein products according to the EU definition (a minimum of 20% energy from protein). Vegetable waste can be a valuable additive for the improvement of the nutritional value of food.

## 1. Introduction

In recent years, the world has been grappling with the growing problem of food waste. Currently, there is worldwide wastage of 1.3 billion tons of food annually, one third of which may be edible. Of this amount, 88 million tons are wasted in the European Union. Most waste is produced by consumers (53%) and producers (19%). Food waste is discussed not only as a phenomenal aspect but also as in terms of its economic, social and energy aspects and has an impact on environmental issues [1,2]. At each stage of the food chain, there is a responsibility for food waste; hence, it is necessary to take actions to reduce these losses. An answer to the problem of food waste is the recently growing zero waste trend. Zero waste can be described as a “set of principles that concentrates on the prevention of waste, which inspires to redesign the life cycle of resources so that all products are reused” [3]. This trend influences all areas of human life, including food production and household management. During the process of vegetable production and consumption, tons of by-products are generated each year, and their storage causes environmental pollution, which can be attributed to their organic composition and moisture content [4]. Waste and by-products from vegetable processing are a large potential source of food proteins that can be used in the design of new food products and in the production of animal feed. The main property of proteins intended for use in food products should be their digestibility, together with the fact that their post-digestion products (peptides) have potential bioactive functions [5].

Everyday food products are increasingly being enriched with high-protein waste products from the plant food industry. One of these products is pasta, due to the ease of its enrichment and its increasing consumption. Pasta protein is an incomplete protein; hence, supplementation of pasta with proteins from other plant sources can improve its nutritional value. The literature provides information on the enrichment of pasta with such waste products as chia seed pomace [6], peanut and carrot waste [7], grape and olive pomace [8], wheat bran and kernel [9,10], hemp seed cake [11], dragonhead seed residue [12], coconut residue [13], and wheat germ [14]. In all of the above-mentioned studies, the addition of by-products or waste from the food industry has resulted in an increase in the protein content in the finished product, demonstrating their high nutritional potential. At the same time, new plant-based additives for pasta and other foods that have not yet been tested are continuously being sought. One of these additives is processing waste from tomato (*Solanum lycopersicum*) and annual pepper (*Capsicum annuum*). Waste from these vegetables accounts for about 40% of their total weight [15,16]. As reported by Nour et al. [17] and Del Valle et al. [18] dried tomato waste (seeds and skin) contains 17–23% protein, while the protein content in the placenta and seeds of peppers is 28.38% and 28.31% d.m., respectively [19]. In addition, all three of these raw materials are good sources of dietary fibre. However, there is currently no information on their use in pasta production. In view of their nutritional value and the increasing of consumption of pasta, their addition in this product appears to be justified.

Given the above, the aim of the study was to determine the possibility of adding tomato and pepper processing waste (tomato waste, pepper placenta, defatted pepper seeds) to durum wheat pasta and to evaluate the physicochemical properties of the resulting products. The research hypothesis is to improve the nutritional value of pasta with the addition of waste from the vegetable industry.

## 2. Materials and Methods

### 2.1. Raw Materials

The following raw materials were used in the study: durum semolina (Julia Malom, Kunszallas, Hungary); by-products from red bell pepper processing, including pepper placenta (PP) and defatted seeds (DPS); and by-products from tomato processing, including tomato waste (TW) (containing seeds and skin) (Krokus, Pająków, Poland). The vegetable wastes were dried for 17 h in an EAC 30-Lab pasta dryer (ItalPast, Fidenza, Italy) using a low temperature profile (30–40 °C) and 72–25% relative humidity of drying air. The pepper seeds were defatted by single extraction with hexane. The by-products were then ground in a laboratory mill (Grindomix GM 200, Retsch, Germany) and stored in plastic bags at a temperature of approximately −18 °C.

### 2.2. Farinograph Characteristics of Mixtures

The mixing properties of semolina with vegetable raw materials at different levels were evaluated by standard Farinograph test, using a Farinograph-E (model 8110142, Brabender, Duisburg, Germany) according to the AACC method [20]. The development time, water absorption, dough stability, degree of dough softening (after 12 min), and Farinograph quality number were analysed.

### 2.3. Fractional Composition of Raw Materials

Determination of the particle size of the semolina durum and vegetable raw materials involved estimating the degree of fragmentation through sieve analysis. To achieve this, a 100 g sample underwent sieving for a duration of 10 min. A sieve shaker (ZBPP, Bydgoszcz, Poland) was employed, utilizing sieves with sizes of 400, 315, 250, 160, 125, and 80 μm. The equivalent diameter was subsequently determined.

### 2.4. Preparation of Pasta Samples

Pasta samples were produced in laboratory conditions on a semi-technical scale using a MAC-30S-Lab extruder (ItalPast, Fidenza, Italy). The level of vegetable waste addition was determined in preliminary studies. In this study, each vegetable raw material was used at the level of 10%, 20%, and 30%. Water was added in a sufficient amount to obtain a mixture with moisture content of 32%. The mixtures were premixed for 15 min. The dough was then extruded under vacuum. The temperature of the extruder barrel did not exceed 28 °C. A Teflon die was used to produce the tagliatelle pasta. In the next step, the pasta was dried for 7 h in an EAC 20-Lab pasta dryer (ItalPast, Fidenza, Italy) at 35–55 °C and 85–55% relative humidity. The samples were then stored at −18 °C. The experiment model and production parameters, such as pressure and extruder capacity, are given in Table 1.

### 2.5. Chemical Composition of Raw Materials and Pasta Samples

The chemical analyses of the raw materials and of the samples of both raw and cooked pasta were performed to determine the following components: protein, fat, dietary fibre, ash, digestible carbohydrates (by difference), moisture, and essential and nonessential amino acids. The enzymatic method, as described by AACC (2000) and AOAC International (2016), was utilized to assay the dietary fibre content. This method enables the determination of the insoluble dietary fibre fraction (IDF), the soluble dietary fibre fraction (SDF), and the total dietary fibre (TDF). The analyses were conducted using enzyme sets and procedures provided by Megazyme, located in Bray, Ireland. The calculation of the total dietary fibre (TDF) involved summing the IDF and SDF fractions. The moisture content was determined using the dryer-weighing method (AACC 44-15A), and the ash content was determined using the AACC 08-01 method. The protein content was determined using a Kjeltec TM8400 instrument (Foss, Copenhagen, Denmark), with a nitrogen-to-protein conversion ratio of 5.70. The total fat content was determined through acid hydrolysis using the continuous extraction method with a SoxtecTM8000 instrument (Foss, Copenhagen, Denmark) and hexane as the solvent. The amino acids containing sulphur were subjected to separate hydrolysis using 6 M HCl. Prior to hydrolysis, oxidation was carried out using a mixture of formic acid and hydrogen peroxide in a volumetric ratio of 9:1. This oxidation process lasted for 20 h at a temperature of 4 ± 1 °C. The determination of amino acids was performed using an AAA 400 amino acid analyser manufactured by INGOS, located in the Czech Republic. Ion exchange chromatography was employed, followed by post-column ninhydrin-based detection using sodium citrate buffer. The ninhydrin derivatives of amino acids were detected at a wavelength of 570 nm for primary amino acids and at 440 nm for secondary amino acids. The digestible carbohydrate content was calculated by subtracting the combined mass of protein, fat, TDF, and ash from 100 in 100 g of dry weight of pasta or raw material. The energy values for the pasta samples were calculated using the modified Atwater coefficient (4 kcal for protein, 4 kcal for carbohydrates, 9 kcal for fat, and 2 kcal for TDF). Determinations of the chemical compositions of the raw materials and the pasta were performed in triplicate.

The chromium content was analysed on an inductively coupled plasma excitation mass spectrometer (ICP Mass Spectrometer MS-820, Varian Inc., Palo Alto, CA, USA). Calcium, magnesium, potassium, and sodium contents were determined using a spectrophotometer (FAAS, Solaar939, Unicam, Cambridge, UK) while zinc content was determined using an ET-AAS spectrophotometer (VARIAN AA 280 FS, Varian Inc., Palo Alto, CA, USA). The phosphorus content was determined with the spectrophotometric method using a Shimadzu UV-1800 spectrophotometer.

### 2.6. Cooking Quality of Pasta Samples

The cooking quality parameters, i.e., the minimum cooking time, weight increase index, and cooking losses, were determined according to [21].

### 2.7. Color Parameters of Pasta Samples

The parameters of the dried pasta samples were determined with the reflectance method based on the Commission Internationale de l’Eclairage L*a*b* colour system, using a spherical spectrophotometer (Chroma Meter CR-5; Konica Minolta, Sakai, Osaka, Japan). The evaluation was made using a standard light source (D65) and a standard colorimetric observer with a field of view of 10°. An 8 mm diameter aperture was used for the measurement. Colour coordinates (L*, a*, b*) were determined according to the CIE system. The spectrophotometer was calibrated using white and black standard plates.

### 2.8. Statistical Analysis

Mean values and standard deviations were calculated. The results were statistically analysed (*p* < 0.05) using one-way analysis of variance with replication (ANOVA, STATISTICA 13, Statsoft).

## 3. Results and Discussion

### 3.1. Physicochemical Properties of Raw Materials

The chemical analysis of the raw materials (Table 2) was performed to determine the content of moisture, ash, protein, fat, carbohydrates (by difference) and soluble (SDF) and insoluble (IDF) dietary fibre. The protein content in the pepper placenta and defatted pepper seeds was 30.77% d.m. and 26.07% d.m., respectively. Adeyeye [19] has reported the protein content of 28.39% d.m. in pepper placenta and 28.31% in non-defatted seeds. All the raw materials analysed in this study are good sources of dietary fibre. The pepper placenta contained almost two-fold lower levels of fibre than defatted pepper seeds and tomato waste but contained almost the same amount of TDF as SDF, making it a very good raw material for soluble fibre enrichment, which is often deficient in the human diet. The fat content was determined to be 3.15% d.m. in the pepper placenta and 10.40% in the defatted pepper seeds. Before defatting, the seeds contained 26.01% d.m. of fat (data not provided), which is similar to the results reported by Cvetković et al. [22]. The tomato waste contained 24.56% d.m. of protein, 11.73% d.m. of fat, and 3.76% d.m. of ash.

The ash content was 0.87% d.m. in the semolina and significantly higher in the other raw materials, i.e., 3.49% d.m. in DPS and 13.29% d.m. in PP. In the study conducted by [19] the content of this ingredient in non-defatted pepper seeds and placenta was 4.14% d.m. and 9.66% d.m., respectively. The content of all the minerals determined in the present study was higher in the vegetable raw materials than in the semolina (Table 2 and Table 3). Studies have shown that PP is a good source of K (36.43 mg g^−1^) (Table 3), which has positive effects on the human cardiovascular system [23]. In turn, pepper seeds are low in sodium and rich in zinc, which is beneficial to the nervous system, immune system, and skin, but its deficiency is increasingly being observed in society [24]. The analysed pepper seeds also had higher Cr content (6.35 mg g^−1^) than the other raw materials. The vegetable raw materials studied were also found to be a better source of Ca than the semolina. The Ca content of tomato waste was 1.3 mg g^−1^, which is in line with the results reported by Nour et al. [17].

The sieve analysis of the utilized raw materials is presented in Table 4. In the case of semolina, the dominant fractions were 315–400 µm and 160–250 µm (with an equivalent diameter of 264.38 µm). The highest proportion of the largest fraction (>400 µm) was found in defatted pepper seeds (61.91%). Tomato waste also exhibited a higher particle size distribution, with 48.47% of the fraction falling within the 315–400 µm range. On the other hand, pepper placenta contained a higher proportion, with fractions of 160–250 µm and 125–160 µm, indicating a finer particle size compared with semolina.

### 3.2. Farinograph Parameters

Determination of the farinographic characteristics of flour can help to design a new product recipe. Its results make it easier to manage the parameters of the production process and provide an answer to the question of the impact of a specific additive on the water absorption of flour and consistency of the dough. Table 5 gives the basic farinographic characteristics of semolina and semolina mixtures with the vegetable components. The development time of CON was 5.15 min but increased significantly in samples enriched with PP and TW, which is probably related to the high fibre content in the mixtures. This indicates that a longer dough mixing time should be used in the case of enrichment with these raw materials. Fortification of the semolina caused an increase in water absorption. The highest values were exhibited by the TW-fortified samples. The level of water absorption in CON was 54.1%, a result similar to that reported by Welc-Stanowska et al. [25]. Water absorption also depends on the fibre content in the mixture. The addition of fibre contributes to a greater ability of the mixture to absorb water by hindering water absorption by starch.

The dough stability was 12.53 min in CON and decreased in the TW- and DPS-enriched samples. An increase in this parameter was observed in the PP20 and PP30 samples (20.46 and 39.30 min, respectively). This may have been caused by the formation of gluten–phenolic acid complexes. During dough formation, hydrogen bonds may form between the polypeptide chain and the hydroxyl group of phenolic acids present in pepper placenta and between the polypeptide chain and the oxygen of the carboxyl group. This may also be the cause of the low softening value of the dough [26]. A similar decreasing trend was noted in the TW samples, which may have had similar causes. The opposite trend in bread dough fortified with pepper placenta has been noted by Jasna et al. [27]. The literature reports that the farinographic characteristics of dough may also be influenced by the content of individual minerals. Magnesium ions present in PP (Table 3) can trigger reactions between proteins increasing dough resistance to mixing and softening [28]. The gluten matrix may also be affected by the potassium and sodium content, with a similar effect on dough stability, as suggested by Abedi and Pourmohammadi [29].

### 3.3. Pasta Processing

The development of a new pasta formulation may cause various problems related to the correctness of the technological process, which is why preliminary studies are necessary. The results of this study (data not provided) show that the 30% supplementation of the pasta with the selected waste raw materials was the maximum addition level that did not interfere with extrusion. Table 1 shows the research model and such basic process parameters as extrusion pressure and extruder capacity. The highest extrusion pressure was obtained for the CON sample (12.95 MPa). Each addition of vegetable raw materials resulted in a decrease in the extrusion pressure, compared with CON. Statistically significant differences were recorded for the DPS20, DPS30 (defatted pepper seed pasta), PP20, PP30 (pepper placenta pasta), and TW30 (tomato waste pasta) samples. The lowest value was recorded for sample PP30 (12.25 MPa). The extruder capacity also decreased with the fortification, but these differences were not statistically significant (*p* < 0.05) in most cases. Albumins are the main tomato waste proteins [30], while globulins are the major bell pepper proteins [31]; both types of proteins can significantly affect extrusion parameters by weakening the gluten matrix. This, in turn, may be related to the enzymatic activity of these proteins. In the case of the TW additive, its high fat content may have induced the reduction in the extrusion pressure and capacity (Table 1).

### 3.4. Chemical Composition of Pasta Samples

The pasta samples were subjected to the analysis of the chemical composition (Table 6). Already at the 10% addition, the fortification with all the three vegetable additives caused a statistically significant increase in the protein content of the pasta from 17.26% d.m. in DPS10 to 20.61% d.m. in TW30, which is an increase of 6 to 27%. A similar relationship was noted for the ash content, where each fortification variant also resulted in a statistically significant increase. The highest increase in the ash content in the pasta sample was recorded in the PP-fortified samples, which was related to the high content of this ingredient in the raw material (13.39% d.m.). In a study conducted by Ahmad et al. [32] the addition of 8% of tomato waste to cookies resulted in a 23% d.m. increase in the protein content. In our study, the addition of 10% of the tomato waste increased the protein content in the sample by 17%.

The fat content in the fortified samples increased in relation to the CON sample, but these differences were not statistically significant for the DPS10, PP20, and TW10 samples. The largest (almost four-fold) increase in this macronutrient was recorded for sample TW30 (4.54% d.m.). The fat contained in the waste raw materials tested consists mainly of unsaturated fatty acids [33,34], which are expected to be present in the diet but may reduce the shelf life of the finished product due to the oxidative processes involved [35].

The addition of PP, DPS, and TW had a significant effect on the TDF content of the samples as well. Each fortification variant caused a statistically significant increase in this parameter (*p* < 0.05). The smallest increase was observed in the pepper placenta-enriched samples, while very similar 5.90-fold and 5.36-fold increase were observed in samples DPS30 (defatted pepper seeds) and TW30 (tomato waste), respectively, which is consistent with the fibre content in the raw materials. Therefore, it can be concluded that DPS and TW are better fibre raw materials than PP; of note, the highest soluble fibre content was recorded in the PP-supplemented samples (8.77% d.m. in PP30). However, all the samples can be considered high-fibre products (TDF content > 6 g/100 g of the pasta samples).

Given the increased protein and TDF content in the experimental pasta samples compared with CON, the carbohydrate content decreased, and the decrease was statistically significant in all the samples (*p* < 0.05). It ranged from 10.10% for PP10 to as much as 62.22% and 57.36% for TW30 and DPS30, respectively. A similar relationship has been reported for pasta samples fortified with wheat germ and wheat germ protein isolate in a study conducted by Teterycz et al. [14].

The energy value in CON was 391.5 kcal/100 g, and this result was similar to that obtained by [11], i.e., 397.39 kcal/100 g. The calorific value of the samples enriched with defatted pepper seeds, pepper placenta, and tomato waste decreased significantly (*p* < 0.05) in each fortification variant. The smallest decrease of 13.42 and 17.93 kcal/100 g was recorded in the PP10 and DPS10 samples, respectively, while the largest decrease of 38.69 kcal/100 g was recorded in the DPS30 sample. In our research, the higher fibre content, with a calorie value of only 2 kcal/g, was responsible for the lower energy value of the product.

The research showed that the six samples tested could be classified as a high-protein products according to the EU definition [36]. This definition states that, in a high-protein product, a minimum of 20% of the energy comes from protein. This criterion was met by samples enriched with 30% DPS, minimum 20% PP and all samples with TW addition. The highest value was obtained for the sample with 30% PP addition (22.65%), which may suggest that it is the best raw material for protein enrichment of food. A similar high-protein pasta was obtained by enrichment of pasta dough with 25% of wheat germ or 12% of wheat germ protein isolate [14] or in samples with 40% addition of hemp seed flour [11].

### 3.5. Mineral Composition of Selected Dried Pasta Samples

The mineral content of selected pasta samples is given in Table 3. Each time, the fortification resulted in a statistically significant increase in the level of minerals relative to CON, which is very important from a nutritional point of view, especially since there has been a decline in the micro- and macronutrient content of vegetable crops over the years [37]. The DPS30 sample exhibited a nearly two-fold increase in the Zn content, an over four-fold increase in the Cr content, and a two-fold increase in the Mg content relative to CON. Sample PP30 had the highest levels of K and Na, which can contribute to the improvement of the rheological parameters of the dough [29], and TW30 contained the highest amount of Ca. Similarly, a study conducted by [38] reported a significant increase in the Ca content in bread fortified with tomato waste.

### 3.6. Amino Acid Composition in Raw Materials and Pasta Samples

To be able to fully evaluate a product with high protein content, it is necessary to assess the amino acid composition. For this purpose, the amino acid composition of a product is often compared with the reference composition given by the WHO [39]. In this study, the amino acid composition was determined for the raw materials and pasta samples (Table 7 and Table 8). Among the raw materials, DPS had the best amino acid composition, where all essential amino acids were present at an appropriate level. The main limiting amino acids were lysine and tryptophan in the durum semolina, histidine in the pepper placenta, and tryptophan in the tomato waste. Nevertheless, the lysine content in the vegetable raw materials was about twice that of semolina (21.78 mg g^−1^ protein) and can therefore improve the quality of wheat protein. In addition, these raw materials are a very good source of sulphur amino acids (Met + Cys) (123.25 mg g^−1^ protein in DPS) and Asp (215 mg g^−1^ protein in pepper placenta), which is also very promising. However, the vegetable raw materials contained less Glu and Pro than the semolina.

The analysis of the amino acid composition showed that the enrichment of the pasta with the vegetable processing by-products improved the overall protein quality. Of particular note are lysine and threonine, which are the limiting amino acids of wheat protein. An increase in the content of lysine was observed in each enriched sample, with the largest increase (by as much as 58%) observed in the TW30 sample. The threonine content increased as well. According to the WHO, the threonine content should be 23 mg per 1 g of protein, which was achieved in samples PP30 and TW30, while the standard content of lysine is 45 mg per 1 g. Unfortunately, lysine was still the limiting amino acid in the samples. The standard amino acid composition was not achieved when pasta was enriched with *Hibiscus sabdariffa* by-product powder [40]. Nevertheless, the addition of vegetable waste significantly improves the amino acid composition in pasta.

### 3.7. Cooking Quality of Pasta Samples

**Figure 1:**
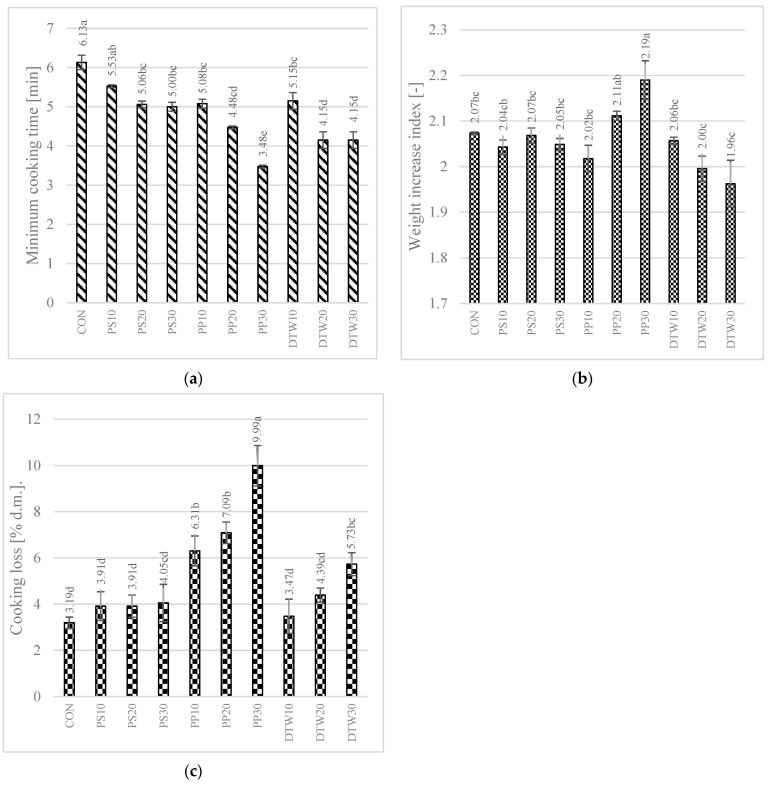
Cooking quality of pasta samples. (**a**) minimum cooking time; (**b**) weight increase index; and (**c**) cooking loss. CON—control sample (100% semolina durum pasta); DPS—defatted pepper seed pasta; PP—pepper placenta pasta; TW—tomato waste pasta. Data are presented as mean (n = 3) ± standard deviation, means on the same bar with different letters are significantly different (Tukey test; *p* ≤ 0.05).

Consumers pay a great deal of attention to the behaviour of pasta during cooking; therefore, a cooking quality evaluation (Figure 1) of the product should be carried out before marketing. One of the most important quality determinants is the minimum cooking time. A shorter cooking time is preferred by the consumer. The study showed that the minimum cooking time decreased with the addition of the vegetable processing by-products, with the greatest decrease recorded in the pepper placenta-enriched samples (3.48 min for PP30), compared with CON (6.13 min).

The water absorption index (WAI) is an indicator of the ability of the product to bind water during cooking. Pasta with good properties should have a WAI of at least 2 [10]. It is recognised that the increase in the WAI value is directly proportional to the pasta cooking time [41]. Starch has the highest water absorption capacity, but the literature also indicates this property in, e.g., pectin, polyphenols, and dietary fibre content [42]. Statistically significant changes in this parameter in comparison with CON (2.07) were recorded only in sample PP30 (2.19), where an increase in WAI was recorded despite the shortest cooking time. This may have been related to the high pectin content of the raw material used [43]. Additionally, the polyphenols present in bell pepper [44] may have interacted with protein, starch, and other polysaccharides, affecting the water absorption of the product [45].

Cooking losses should not exceed 8%. An increase in this parameter was recorded in all the samples, with statistical significance in all of the pepper placenta-fortified samples. Despite the highest WAI value, sample PP30 exhibited the highest cooking loss value. This may have been influenced by the high fibre content, including soluble fibre, and the high degree of milling of the raw material (Table 4). Equivalent diameter—indicating particle size—for pepper placenta was significantly lower than for semolina, facilitating the passage of particles into the water during cooking. For the other raw materials, this parameter was higher compared to semolina. Only for sample PP30 was the optimum value of 8% (9.99%) exceeded. Cooking losses are dependent not only on the chemical composition of the product but also on its shape [46]. This shape (tagliatelle) gives a large contact area between the pasta and the water, which may also cause a higher level of dry matter losses.

### 3.8. Colour Parameters of Dried Pasta Samples

**Figure 2:**
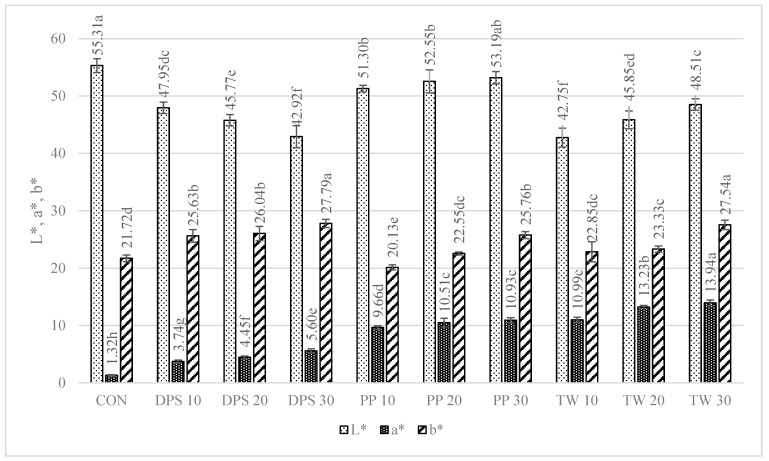
Colour parameters of dried pasta samples. CON—control sample (100% semolina durum pasta); DPS—defatted pepper seed pasta; PP—pepper placenta pasta; TW –tomato waste pasta. L*: lightness; a*: red/green value; b*: blue/yellow value. Data are presented as mean (n = 30) ± standard deviation, means on the same bar with different letters are significantly different (Tukey test; *p* ≤ 0.05).

The colour of the pasta is the first characteristic evaluated by the consumer during purchase, and it is important that it is perceived positively. The study measured the parameters of the pasta after drying (Figure 2). The analysis of the L* parameter indicated significant (*p* < 0.05) darkening of the enriched pasta, compared with CON, in all samples. CON was the brightest sample (55.31). The lowest L* parameters were exhibited by the TW-enriched samples (42.75–48.51) and the DPS fortification variants (47.95–42.92), while the pepper seed-enriched samples had the highest values of this parameter (51.30–53.19). The PP- and TW-supplemented samples exhibited higher values of the brightness parameter. This may be related to the presence of non-hydrated bright fibre particles resulting from coarser particle granulation (Table 4), as can be seen in the photos presented in Table 9. TW and PP are good sources of carotenoids, mainly capsanthin in pepper [47] and lycopene in tomatoes [48], which were found to make the colour of the product darker than the CON sample. The content of these ingredients undoubtedly affected the a* parameter as well. It is responsible for the colour change from green to red. In the case of the pasta enriched with PP and TW, the a* parameter exhibited an over 8-fold increase in sample PP30 (10.93) and an up to 10-fold increase in sample TW30 (13.94), compared with CON (1.32). The a* parameter also increased in the pepper seed-fortified samples and reached 3.74 in DPS10 to 5.60 in DPS30.

In comparison with CON, statistically significant differences in the b* parameter (blue-yellow) were noted in all samples, except PP10, PP20, and TW10. Considering all colour parameters, the DPS-supplemented pasta became dark yellow, while the pasta enriched with PP and tomato waste became intensely orange (Table 9). A similar trend was noted for pasta fortified with bell pepper [49] and for tomato pomace-enriched bread [50].

## 4. Conclusions

The utilization of food processing waste, including vegetable processing waste, appears to be a promising direction for research. This trend enables the creation of new products with novel organoleptic and nutritional properties, while also offering an opportunity to reduce the food waste that has become such a global issue. This study has demonstrated that both tomato and pepper processing waste serve as excellent sources of protein and fibre, which can enhance the nutritional value of cereal products, including pasta. The samples supplemented with tomato waste exhibited the highest protein content and were also rich in fibre. Pasta enriched with a minimum of 20% pepper placenta, at least 30% defatted pepper seeds, and a minimum of 10% tomato waste represents a high-protein and high-fibre product with favourable cooking properties. Despite fortifying the pasta with high-protein raw materials, achieving a balanced protein profile was not possible as lysine remained the limiting amino acid in the samples.

Future research will be extended to include texture analysis, sensory evaluation and digestibility assessment.

## Figures and Tables

**Table 1:** Model of experiment and pasta processing parameters.

Samples	Pasta Formula				Production Parameters	
	Semolina	Defatted Pepper Seeds	Pepper Placenta	Tomato Waste	Pressure	Extruder Capacity
	[%]				[MPa]	[kg/h]
**CON**	**100**				12.95 ^a^ ± 0.71	31.06 ^a^ ± 0.07
**DPS 10**	90	10			12.85 ^ab^ ± 2.12	30.78 ^a^ ± 0.08
**DPS 20**	80	20			12.35 ^d^ ± 0.71	30.30 ^ab^ ± 0.08
**DPS 30**	70	30			12.45 ^cd^ ± 0.71	30.16 ^abc^ ± 0.20
**PP 10**	90		10		12.75 ^abc^ ± 0.71	29.94 ^abc^ ± 0.39
**PP 20**	80		20		12.45 ^dc^ ± 0.71	29.01 ^bc^ ± 0.92
**PP 30**	70		30		12.25 ^d^ ± 0.71	28.93 ^bc^ ± 0.16
**TW 10**	90			10	12.95 ^a^ ± 0.71	30.77 ^a^ ± 0.27
**TW 20**	80			20	12.75 ^abc^ ± 0.71	29.76 ^abc^ ± 0.51
**TW 30**	70			30	12.55 ^bcd^ ± 0.71	28.56 ^b^ ± 0.51

CON—control sample (100% semolina durum pasta), DPS—defatted pepper seeds pasta; PP—pepper placenta pasta; TW—tomato waste pasta. Means (n = 3) with different letters (a–d) in the same column are significantly different (Tukey test; *p* ≤ 0.05).

**Table 2:** Chemical properties of raw materials.

Raw Materials	Moisture	Ash	Protein	Fat	Carbohydrates *	TDF	IDF	SDF
	%	% d.m.						
Semolina durum	8.45 ^b^ ± 0.28	0.87 ^d^ ± 0.07	16.19 ^d^ ± 0.08	1.24 ^d^ ± 0.01	75.85 ^a^ ± 0.07	4.74 ^c^ ± 0.11	3.51 ^d^ ± 0.11	1.27 ^d^ ± 0.05
Defatted pepper seeds	5.96 ^c^ ± 0.01	3.49 ^c^ ± 0.04	26.07 ^b^ ± 0.06	10.40 ^b^ ± 0.21	2.32 ^c^ ± 0.08	60.89 ^a^ ± 0.13	53.71 ^a^ ± 0.12	7.19 ^c^ ± 0.25
Pepper placenta	11.84 ^a^ ± 0.38	13.29 ^a^ ± 0.13	30.77 ^a^ ± 0.01	3.15 ^c^ ± 0.04	19.28 ^b^ ± 0.39	33.49 ^b^ ± 0.55	16.86 ^c^ ± 1.56	16.63 ^a^ ± 1.01
Tomato waste	6.41 ^c^ ± 0.08	3.76 ^b^ ± 0.01	24.56 ^c^ ± 0.02	11.73 ^a^ ± 0.04	3.11 ^c^ ± 0.32	60.92 ^a^ ± 0.37	49.72 ^b^ ± 0.36	11.20 ^b^ ± 0.01

* carbohydrate content calculated by difference. IDF—insoluble dietary fibre. SDF—soluble dietary fibre. TDF—total dietary fibre. Means (*n* = 3) with different letters in the same column are significantly different (Tukey test; *p* ≤ 0.05).

**Table 3:** Mineral composition of raw materials and selected dried pasta samples.

Samples	P	Ca	Mg	K	Cr	Na	Zn
	mg/g	mg/g	mg/g	mg/g	µg/g	µg/g	µg/g
	**Raw materials**						
Semolina durum	2.20 ^d^ ± 0.02	0.19 ^d^ ± 0.03	0.59 ^d^ ± 0.08	2.33 ^d^ ± 0.04	0.44 ^d^ ± 0.05	nd	11.41 ^d^ ± 0.16
Defatted pepper seeds	7.07 ^a^ ± 0.02	0.86 ^c^ ± 0.08	2.79 ^a^ ± 0.04	7.35 ^c^ ± 0.06	6.35 ^a^ ± 0.04	23.5 ^b^ ± 0.01	39.59 ^a^ ± 0.11
Pepper placenta	3.58 ^c^ ± 0.05	1.02 ^b^ ± 0.07	1.19 ^c^ ± 0.04	36.43 ^a^ ± 0.11	1.46 ^c^ ± 0.06	115.00 ^a^ ± 4.24	23.56 ^c^ ± 0.08
Tomato waste	5.14 ^b^ ± 0.03	1.39 ^a^ ± 0.05	2.27 ^b^ ± 0.02	10.69 ^b^ ± 0.08	3.06 ^b^ ± 0.08	113.00 ^a^ ± 2.83	25.66 ^b^ ± 0.08
	**Pasta samples**						
CON	2.23 ^d^ ± 0.02	0.23 ^d^ ± 0.02	0.59 ^d^ ± 0.04	2.31 ^d^ ± 0.01	0.45 ^d^ ± 0.09	nd	11.38 ^d^ ± 0.05
DPS 30	3.76 ^a^ ± 0.04	0.35 ^c^ ± 0.08	1.22 ^a^ ± 0.03	3.87 ^c^ ± 0.06	1.93 ^a^ ± 0.04	8.50 ^c^ ± 0.05	20.80 ^a^ ± 0.04
PP 30	2.65 ^c^ ± 0.06	0.47 ^b^ ± 0.06	0.72 ^c^ ± 0.07	11.49 ^a^ ± 0.10	0.91 ^c^ ± 0.02	41.00 ^a^ ± 0.28	17.31 ^b^ ± 0.07
TW 30	3.10 ^b^ ± 0.02	0.53 ^a^ ± 0.09	1.04 ^b^ ± 0.04	4.89 ^b^ ± 0.10	1.34 ^b^ ± 0.04	31.70 ^b^ ± 0.28	16.88 ^c^ ± 0.06

CON—control sample (100% semolina durum pasta); DPS—defatted pepper seed pasta; PP—pepper placenta pasta; TW—tomato waste pasta; nd—not detected. Data are presented as mean (n = 3) ± standard deviation, means in the same column (raw material or sample) with different letters are significantly different (Tukey test; *p* ≤ 0.05).

**Table 4:** Fractional composition of raw materials.

Raw Materials	Fractions (µm)							Equivalent Diameter (µm)
	>400	315–400	250–315	160–250	125–160	80–125	<80	
	(%)							
Semolina durum	9.17 ^b^ ± 0.15	27.58 ^b^ ± 0.36	21.83 ^a^ ± 0.26	22.52 ^d^ ± 0.23	6.36 ^b^ ± 0.24	11.48 ^c^ ± 0.04	0.51 ^c^ ± 0.04	264.38 ^c^ ± 1.06
Defatted pepper seeds	61.91 ^a^ ± 0.44	10.75 ^c^ ± 0.17	5.49 ^c^ ± 0.08	17.84 ^c^ ± 0.39	0.30 ^d^ ± 0.09	1.97 ^c^ ± 0.09	1.06 ^c^ ± 0.09	341.28 ^a^ ± 0.64
Pepper placenta	1.55 ^d^ ± 0.15	8.41 ^d^ ± 0.41	14.86 ^b^ ± 0.52	28.27 ^b^ ± 1.02	23.35 ^a^ ± 0.23	20.5 ^b^ ± 0.21	1.75 ^b^ ± 0.21	191.73 ^d^ ± 0.74
Tomato waste	3.86 ^c^ ± 0.24	48.47 ^a^ ± 0.26	5.96 ^c^ ± 0.10	37.79 ^a^ ± 0.61	2.29 ^c^ ± 0.39	1.12 ^a^ ± 0.15	3.05 ^a^ ± 0.15	287.66 ^b^ ± 0.79

Data are presented as mean (n = 3) ± standard deviation, means in the same column (a–d) with different letters are significantly different (Tukey test; *p* ≤ 0.05).

**Table 5:** Farinograph characteristics of mixtures.

Samples	Development Time	Water Absorption	Dough Stability	Degree of Dough Softening (after 12 min)	Farinograph Quality Number
	min	%	min	FU	Mm
CON	5.17 ^e^ ± 0:07	56.60 ^f^ ± 0.14	12.53 ^c^ ± 0:05	35.50 ^c^ ± 0.71	149.50 ^e^ ± 0.71
DPS 10	4.44 ^f^ ± 0:09	56.55 ^f^ ± 0.21	10.20 ^d^ ± 0:13	35.50 ^c^ ± 0.16	138.00 ^e^ ± 2.12
DPS 20	4.39 ^f^ ± 0:21	57.55 ^e^ ± 0.04	5.19 ^g^ ± 0:02	34.50 ^c^ ± 1.41	100.00 ^f^ ± 2.83
DPS 30	5.32 ^e^ ± 0:09	58.15 ^d^ ± 0.04	4.16 ^h^ ± 0:08	19.00 ^f^ ± 0.71	255.50 ^b^ ± 3.54
PP 10	6.48 ^d^ ± 0:17	56.15 ^f^ ± 0.07	7.39 ^f^ ± 0:06	32.11 ^cd^ ± 0.71	104.50 ^f^ ± 1.41
PP 20	10.39 ^b^ ± 0:03	57.33 ^e^ ± 0.07	20.46 ^b^ ± 0:04	28.00 ^d^ ± 0.71	238.00 ^c^ ± 1.41
PP 30	15.40 ^a^ ± 0:02	58.78 ^d^ ± 0.07	39.20 ^a^ ± 0:06	12.50 ^g^ ± 1.41	352.50 ^a^ ± 6.36
TW 10	5.40 ^e^ ± 0:16	61.00 ^c^± 0.28	7.17 ^f^ ± 0:04	46.50 ^a^ ± 2.12	104.50 ^f^ ± 2.12
TW 20	6.20 ^d^ ± 0:01	63.65 ^b^ ± 0.07	5.18 ^g^ ± 0:14	44.50 ^a^ ± 0.71	101.00 ^f^ ± 7.07
TW 30	8.29 ^c^ ± 0:06	64.65 ^a^ ± 0.07	8.30 ^e^ ± 0:10	23.50 ^e^ ± 0.71	208.00 ^d^ ± 1.41

CON—control sample (100% semolina durum pasta), DPS—defatted pepper seeds pasta; PP—pepper placenta pasta; TW—tomato waste pasta. Data are presented as mean (n = 3) ± standard deviation, means in the same column with different letters are significantly different (Tukey test; *p* ≤ 0.05).

**Table 6:** Chemical composition of pasta samples.

Pasta Samples	Moisture	Ash	Protein	Fat	TDF	IDF	SDF	Carbohydrates *	Energy	Energy from Protein	Energy from Protein
	%	% d.m.							kcal/100 g d.m.		%
CON	9.45 ^b^ ± 0.02	1.03 ^g^ ± 0.01	16.16 ^h^ ± 0.08	1.11 ^g^ ± 0.08	4.74 ^f^ ± 0.1	2.94 ^h^ ± 0.06	1.81 ^g^ ± 0.04	76.95 ^a^ ± 0.17	391.95 ^a^ ± 0.22	64.64 ^g^ ± 0.31	16.49 ^f^ ± 0.09
DPS 10	9.21 ^c^ ± 0.02	1.48 ^f^ ± 0.02	17.26 ^g^ ± 0.03	1.38 ^fg^ ± 0.03	13.48 ^cd^ ± 0.23	8.60 ^e^ ± 0.02	4.87 ^f^ ± 0.25	66.44 ^c^ ± 0.24	374.02 ^bc^ ± 0.64	69.04 ^f^ ± 0.12	18.46 ^e^ ± 0.07
DPS 20	9.07 ^c^ ± 0.04	1.67 ^e^ ± 0.04	17.42 ^fg^ ± 0.06	2.52 ^d^ ± 0.05	20.20 ^b^ ± 0.38	14.34 ^c^ ± 0.4	5.86 ^cd^ ± 0.03	58.19 ^d^ ± 0.32	365.52 ^d^ ± 1.19	69.68 ^f^ ± 0.25	19.06 ^d^ ± 0.05
DPS 30	8.73 ^d^ ± 0.03	1.99 ^c^ ± 0.02	17.68 ^f^ ± 0.02	3.44 ^b^ ± 0.05	27.99 ^a^ ± 1.05	20.69 ^a^ ± 0.76	7.30 ^b^ ± 0.29	48.90 ^e^ ± 1.14	353.26 ^e^ ± 1.95	70.72 ^e^ ± 0.09	20.02 ^c^ ± 0.14
PP 10	8.86 ^d^ ± 0.03	1.79 ^d^ ± 0.03	17.87 ^e^ ± 0.12	0.99 ^g^ ± 0.01	9.63 ^e^ ± 0.46	4.31 ^gh^ ± 0.11	5.32 ^def^ ± 0.05	69.72 ^b^ ± 0.04	378.53 ^b^ ± 0.39	71.48 ^d^ ± 0.47	18.88 ^e^ ± 0.11
PP 20	9.51 ^b^ ± 0.07	2.56 ^b^ ± 0.03	18.55 ^d^ ± 0.06	1.16 ^ef^ ± 0.04	12.25 ^d^ ± 0.25	5.08 ^fg^ ± 0.51	7.17 ^b^ ± 0.05	65.48 ^c^ ± 0.39	371.06 ^c^ ± 1.24	74.20 ^c^ ± 0.25	20.00 ^c^ ± 0.07
PP 30	10.74 ^a^ ± 0.07	3.33 ^a^ ± 0.01	19.24 ^cd^ ± 0.05	1.29 ^e^ ± 0.04	14.57 ^c^ ± 0.16	5.80 ^f^ ± 0.21	8.77 ^a^ ± 0.03	61.57 ^d^ ± 0.15	363.99 ^d^ ± 0.65	76.96 ^b^ ± 0.19	21.14 ^b^ ± 0.01
TW 10	9.14 ^c^ ± 0.07	1.56 ^e^ ± 0.01	18.96 ^c^ ± 0.09	1.38 ^fg^ ± 0.05	13.54 ^cd^ ± 0.28	8.24 ^e^ ± 0.34	5.29 ^ef^ ± 0.05	64.56 ^c^ ± 0.26	373.58 ^b^ ± 0.54	75.84 ^c^ ± 0.34	20.30 ^c^ ± 0.06
TW 20	8.73 ^d^ ± 0.08	1.77 ^d^ ± 0.03	19.25 ^b^ ± 0.09	2.95 ^c^ ± 0.05	18.56 ^b^ ± 0.01	12.87 ^d^ ± 0.16	5.70 ^de^ ± 0.17	57.47 ^d^ ± 0.01	370.55 ^c^ ± 0.14	77.00 ^b^ ± 0.34	20.78 ^b^ ± 0.14
TW 30	8.41 ^e^ ± 0.05	1.88 ^d^ ± 0.01	20.61 ^a^ ± 0.08	4.54 ^a^ ± 0.05	25.44 ^a^ ± 0.22	19.04 ^b^ ± 0.14	6.40 ^c^ ± 0.08	47.43 ^e^ ± 0.27	363.90 ^d^ ± 0.53	82.44 ^a^ ± 0.31	22.65 ^a^ ± 0.11

* Carbohydrate content calculated by difference. CON—control sample (100% semolina durum pasta); DPS—defatted pepper seeds pasta; PP—pepper placenta pasta; TW—tomato waste pasta; IDF—insoluble dietary fibre; SDF—soluble dietary fibre; TDF—total dietary fibre. Data are presented as mean (n = 3) ± standard deviation, means in the same column with different letters are significantly different (Tukey test; *p* ≤ 0.05).

**Table 7:** Non-essential amino acid composition of raw materials and dried pasta samples (mg g−1 protein).

Amino Acid Scoring Pattern for Adults *							
Amino Acid	Ala	Asp	Glu	Gly	Pro	Ser	Arg
**Raw materials**							
Semolina durum	31.55 ^c^ ± 0.41	49.04 ^d^ ± 0.49	301.27 ^a^ ± 0.09	28.54 ^c^ ± 0.44	115.29 ^a^ ± 0.83	45.97 ^a^ ± 0.06	36.97 ^c^ ± 0.15
Defatted pepper seeds	43.69 ^a^ ± 0.04	100.13 ^c^ ± 0.18	195.94 ^d^ ± 0.37	48.35 ^a^ ± 0.42	48.06 ^b^ ± 0.01	42.52 ^b^ ± 0.08	83.90 ^a^ ± 0.50
Pepper placenta	14.50 ^d^ ± 0.26	215.48 ^a^ ± 0.19	219.83 ^b^ ± 0.75	12.61 ^d^ ± 0.28	12.32 ^d^ ± 0.33	32.60 ^d^ ± 0.25	21.61 ^d^ ± 0.05
Tomato waste	35.14 ^b^ ± 0.04	114.88 ^b^ ± 1.04	202.35 ^c^ ± 0.29	44.16 ^b^ ± 0.03	45.73 ^c^ ± 0.03	41.04 ^c^ ± 0.03	62.75 ^b^ ± 0.35
**Pasta samples**							
CON	31.68 ^bc^ ± 0.42	49.16 ^g^ ± 0.48	301.39 ^a^ ± 0.09	28.66 ^ef^ ± 0.43	115.41 ^a^ ± 0.84	46.10 ^a^ ± 0.07	37.09 ^f^ ± 0.15
DPS 10	31.64 ^bc^ ± 0.55	47.15 ^g^ ± 0.20	267.15 ^ed^ ± 3.80	27.34 ^gh^ ± 0.12	103.78 ^b^ ± 3.52	42.87 ^c^ ± 0.24	39.09 ^ed^ ± 0.10
DPS 20	33.05 ^ab^ ± 0.04	56.96 ^f^ ± 0.31	250.51 ^f^ ± 3.47	32.85 ^b^ ± 0.23	90.40 ^d^ ± 0.31	42.10 ^d^ ± 0.19	43.45 ^b^ ± 0.34
DPS 30	33.98 ^a^ ± 0.04	63.45 ^d^ ± 0.11	235.01 ^g^ ± 0.15	34.08 ^a^ ± 0.07	81.25 ^e^ ± 0.44	40.88 ^e^ ± 0.37	49.06 ^a^ ± 0.11
PP 10	30.24 ^cde^ ± 0.08	77.78 ^c^ ± 1.02	282.86 ^b^ ± 1.05	27.95 ^fg^ ± 0.53	97.57 ^c^ ± 0.49	43.80 ^b^ ± 0.08	31.04 ^g^ ± 0.02
PP 20	28.40 ^f^ ± 0.19	101.05 ^b^ ± 0.45	274.79 ^c^ ± 0.33	26.42 ^h^ ± 0.15	78.11 ^e^ ± 0.07	40.40 ^e^ ± 0.22	30.92 ^g^ ± 0.10
PP 30	28.76 ^ef^ ± 0.41	133.26 ^a^ ± 0.55	260.94 ^e^ ± 0.59	25.07 ^i^ ± 0.41	61.64 ^f^ ± 0.62	38.12 ^f^ ± 0.21	30.38 ^g^ ± 0.24
TW 10	29.75 ^def^ ± 0.16	57.27 ^f^ ± 0.16	277.28 ^bc^ ± 2.32	29.35 ^de^ ± 0.08	101.52 ^bc^ ± 1.83	38.68 ^f^ ± 0.08	38.41 ^e^ ± 0.22
TW 20	30.75 ^cd^ ± 1.17	60.32 ^e^ ± 0.84	270.42 ^cd^ ± 0.72	30.34 ^d^ ± 0.20	90.10 ^d^ ± 1.21	38.08 ^f^ ± 0.16	39.62 ^d^ ± 0.52
TW 30	29.86 ^cdef^ ± 0.26	61.42 ^de^ ± 0.68	246.4 ^f^ ± 0.42	31.60 ^c^ ± 0.26	81.78 ^e^ ± 0.72	37.25 ^g^ ± 0.08	41.10 ^c^ ± 0.11

CON—control sample (100% semolina durum pasta); DPS—defatted pepper seed pasta; PP—pepper placenta pasta; TW—tomato waste pasta, AAA—aromatic amino acids; SAA—sulphur containing amino acid. Data are presented as mean (n = 3) ± standard deviation, means in the same column (raw material or sample) with different letters are significantly different (Tukey test; *p* ≤ 0.05). * World Health Organization/Food and Agriculture Organization/United Nations University (2007) Protein and Amino Acid Requirements in Human Nutrition Report of a Joint WHO/FAO/UNU Expert Consultation. WHO Technical Report Series no. 935. Geneva: WHO.

**Table 8:** Essential amino acid composition of raw materials and dried pasta samples in comparison with amino acid scoring pattern for adults (FAO, 2007) (mg g^−1^ protein).

Amino Acid	His	Ile	Leu	Lys	Thr	Trp	Val	AAA (Phe + Tyr)	SAA (Met + Cys)	Limiting Amino Acid	Amino Acid Score Using the Pattern for Adults
Amino Acid Scoring Pattern for Adults *	15	30	59	45	23	6	39	38	22		
**Raw materials**											
Semolina durum	19.28 ^c^ ± 0.39	35.63 ^a^ ± 0.97	56.82 ^ab^ ± 0.44	21.78 ^c^ ± 0.69	21.26 ^c^ ± 0.06	17.46 ^a^ ± 0.21	44.88 ^a^ ± 0.50	60.79 ^c^± 0.91	31.67 ^c^ ± 0.64	Lys, Thr	48
Defatted pepper seeds	27.23 ^a^ ± 0.01	32.41 ^b^ ± 0.22	60.35 ^a^ ± 0.25	47.42 ^a^ ± 0.34	21.66 ^c^ ± 0.01	14.34 ^b^ ± 0.08	39.48 ^c^ ± 0.31	74.80 ^a^ ± 0.30	123.25 ^a^ ± 5.51	-	
Pepper placenta	13.50 ^d^ ± 0.12	29.50 ^c^ ± 0.19	53.29 ^ab^ ± 4.14	41.89 ^b^ ± 0.93	34.21 ^b^ ± 0.18	6.90 ^c^ ± 0.17	39.67 ^c^ ± 0.07	38.44 ^d^ ± 0.30	82.61 ^b^ ± 0.07	His,	90
Tomato waste	21.89 ^b^ ± 0.09	28.59 ^c^ ± 0.04	50.37 ^b^ ± 0.11	47.90 ^a^ ± 0.45	41.95 ^a^ ± 0.53	3.68 ^d^ ± 0.05	42.76 ^b^ ± 0.31	64.42 ^b^ ± 0.32	83.09 ^b^ ± 2.93	Trp,	61
**Pasta samples**											
CON	19.41 ^ef^ ± 0.39	35.75 ^a^ ± 0.97	56.95 ^b^ ± 0.43	21.90 ^e^ ± 0.68	21.63 ^f^ ± 0.20	17.58 ^a^ ± 0.22	41.4 ^bcd^ ± 0.06	60.91 ^c^ ± 0.91	32.59 ^g^ ± 0.44	Lys	48
DPS 10	20.70 ^c^ ± 0.11	33.77 ^b^ ± 0.26	57.64 ^ab^ ± 0.54	22.67 ^e^ ± 0.44	21.78 ^f^ ± 0.64	16.12 ^bc^ ± 0.29	43.44 ^ab^ ± 0.30	60.84 ^c^ ± 0.74	44.73 ^de^ ± 0.71	Lys	50
DPS 20	22.31 ^b^ ± 0.08	32.08 ^cde^ ± 0.02	58.51 ^ab^ ± 0.06	29.49 ^b^ ± 0.05	21.29 ^f^ ± 0.10	15.85 ^bcd^ ± 0.05	42.26 ^abc^ ± 0.15	63.86 ^b^ ± 0.53	53.51 ^bc^ ± 1.63	Lys	65
DPS 30	23.35 ^a^ ± 0.14	30.79 ^e^ ± 0.33	59.07 ^a^ ± 0.07	33.97 ^a^ ± 0.48	21.07 ^f^ ± 0.06	15.57 ^cd^ ± 0.01	42.05 ^abc^ ± 0.09	66.10 ^a^ ± 0.18	67.69 ^a^ ± 0.64	Lys	75
PP 10	19.06 ^fg^ ± 0.33	33.44 ^bc^ ± 0.27	54.52 ^c^ ± 0.08	22.00 ^e^ ± 0.05	25.42 ^de^ ± 0.04	16.67 ^b^ ± 0.21	39.82 ^cde^ ± 0.24	61.73 ^c^ ± 0.45	40.17 ^f^ ± 0.08	Lys	48
PP 20	18.30 ^gh^ ± 0.37	32.08 ^cde^ ± 0.10	53.44 ^cd^ ± 0.32	23.76 ^de^ ± 0.13	26.17 ^cd^ ± 0.24	15.24 ^de^ ± 0.10	38.08 ^e^ ± 2.04	56.25 ^d^ ± 0.41	50.75 ^c^ ± 0.69	Lys	53
PP 30	17.85 ^h^ ± 0.07	31.38 ^ed^ ± 0.40	51.68 ^e^ ± 0.93	24.67 ^cd^ ± 0.16	27.00 ^c^ ± 0.03	14.22 ^fg^ ± 0.05	39.16 ^de^ ± 0.07	54.85 ^d^ ± 0.07	56.84 ^b^ ± 1.97	Lys	55
TW 10	19.72 ^def^ ± 0.08	33.26 ^bc^ ± 0.41	54.22 ^cd^ ± 0.16	25.94 ^c^ ± 1.02	24.68 ^e^ ± 0.16	14.41 ^ef^ ± 0.25	44.25 ^a^ ± 0.02	60.17 ^c^ ± 0.53	40.65 ^ef^ ± 0.02	Lys	58
TW 20	20.06 ^cd^ ± 0.09	32.51 ^bcd^ ± 0.20	54.55 ^c^ ± 0.24	29.81 ^b^ ± 0.64	28.56 ^b^ ± 0.50	13.45 ^gh^ ± 0.28	43.52 ^ab^ ± 0.70	63.77 ^b^ ± 0.31	45.76 ^d^ ± 2.08	Lys	66
TW 30	20.34 ^cd^ ± 0.01	30.55 ^e^ ± 0.26	52.83 ^de^ ± 0.38	34.84 ^a^ ± 0.40	29.82 ^a^ ± 0.07	12.76 ^h^ ± 0.36	43.13 ^ab^ ± 0.17	64.17 ^ab^ ± 0.06	50.50 ^c^ ± 0.54	Lys	77

CON—control sample (100% semolina durum pasta); DPS—defatted pepper seed pasta; PP—pepper placenta pasta; TW—tomato waste pasta, AAA—aromatic amino acids; SAA—sulphur containing amino acid. Data are presented as mean (n = 3) ± standard deviation, means in the same column (raw material or sample) with different letters are significantly different (Tukey test; *p* ≤ 0.05). * World Health Organization/Food and Agriculture Organization/United Nations University (2007) Protein and Amino Acid Requirements in Human Nutrition Report of a Joint WHO/FAO/UNU Expert Consultation. WHO Technical Report Series no. 935. Geneva: WHO.

**Table 9:** The photos of pasta samples.

CON	DPS10	DPS20	DPS30
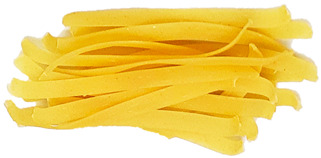	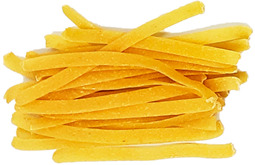	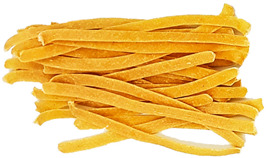	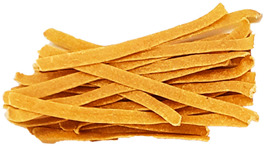
	PP10	PP20	PP30
	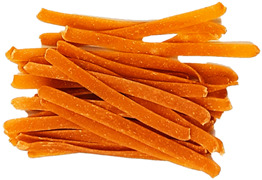	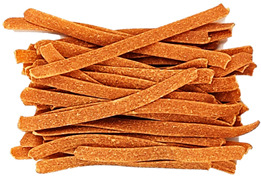	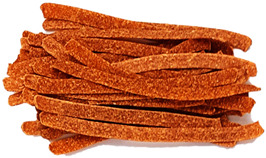
	TW10	TW20	TW30
	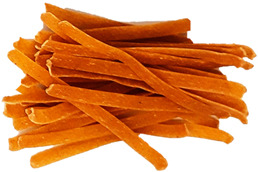	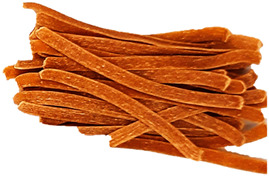	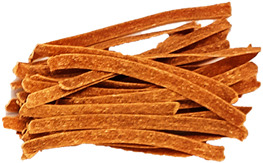

## Data Availability

The data used to support the findings of this study can be made available by the corresponding author upon request.
